# Studies on the Antibacterial Effects of Statins - *In Vitro* and *In Vivo*

**DOI:** 10.1371/journal.pone.0024394

**Published:** 2011-08-30

**Authors:** Peter Bergman, Charlotte Linde, Katrin Pütsep, Anton Pohanka, Staffan Normark, Birgitta Henriques-Normark, Jan Andersson, Linda Björkhem-Bergman

**Affiliations:** 1 Department of Laboratory Medicine, Division of Clinical Microbiology, Karolinska Institutet, Karolinska University Hospital Huddinge, Stockholm, Sweden; 2 Department of Medicine, Centre for Infectious Medicine (CIM), Karolinska Institutet, Karolinska University Hospital Huddinge, Stockholm, Sweden; 3 Microbiology and Tumorbiology Centre (MTC), Karolinska Institutet, Stockholm, Sweden; 4 Clinical Microbiology, Karolinska University Hospital, Solna, Stockholm, Sweden; 5 Department of Laboratory Medicine, Division of Clinical Pharmacology, Karolinska Institutet, Karolinska University Hospital, Huddinge, Stockholm, Sweden; Hannover School of Medicine, Germany

## Abstract

**Background:**

Statin treatment has been associated with a beneficial outcome on respiratory tract infections. In addition, previous *in vitro* and *in vivo* experiments have indicated favorable effects of statins in bacterial infections.

**Aim:**

The aim of the present study was to elucidate possible antibacterial effects of statins against primary pathogens of the respiratory tract.

**Methods:**

MIC-values for simvastatin, fluvastatin and pravastatin against *S. pneumoniae*, *M. catarrhalis* and *H. influenzae* were determined by traditional antibacterial assays. A BioScreen instrument was used to monitor effects of statins on bacterial growth and to assess possible synergistic effects with penicillin. Bacterial growth in whole blood and serum from healthy volunteers before and after a single dose of simvastatin, fluvastatin and penicillin (positive control) was determined using a blood culture system (BactAlert).

**Findings:**

The MIC-value for simvastatin against *S pneumoniae* and *M catarrhalis* was 15 µg/mL (36 mmol/L). Fluvastatin and Pravastatin showed no antibacterial effect in concentrations up to 100 µg/mL (230 µmol/L). Statins did not affect growth or viability of *H influenzae*. Single doses of statins given to healthy volunteers did not affect growth of pneumococci, whereas penicillin efficiently killed all bacteria.

**Conclusions:**

Simvastatin at high concentrations 15 µg/mL (36 µmol/L) rapidly kills *S pneumoniae* and *M catarrhalis*. However, these concentrations by far exceed the concentrations detected in human blood during simvastatin therapy (1–15 nmol/L) and single doses of statins given to healthy volunteers did not improve antibacterial effects of whole blood. Thus, a direct bactericidal effect of statins *in vivo* is probably not the mechanism behind the observed beneficial effect of statins against various infections.

## Introduction

Statins (HMG-CoA reductase inhibitors) are today some of the most prescribed drugs in the world due to their beneficial effects on cardiovascular disease [1]. During recent years statins have been ascribed additional beneficial (pleitropic) effects. This includes anti-inflammatory [2], immunomodulatory [3] and anticarcinogenic properties [4], [5]. In addition, a number of observational studies support that statin treatment is associated with a better prognosis in severe bacterial infections [6], [7]. According to a meta-analysis of these studies patients on statin therapy seem to have a better outcome of bacterial infections (OR 0.53, 95% CI 0.42–0.66). However, the association did not reach statistical significance after adjustment for apparent publication bias OR 0.79 (95% CI 0.58–1.07). The proposed effect of statin seems to be particularly pronounced in respiratory tract infection. Of 15 observational studies on pneumonia and statins, 12 showed that statin-use was associated with a favourable outcome [6].

This proposed beneficial effect of statins might be explained by potential anti-inflammatory properties [2]. In addition, statins have been reported to inhibit host cell invasion by *Staphylococcus aureus* [8] as well as to enhance bacterial clearance of this pathogen [9]. A direct antibacterial effect of statins against *Staphylococcus aureus* has also been proposed [10], [11]. Moreover, statins have been shown to protect against pneumococcal infection in a mouse model of sickle cell disease [12]. Recently, statins were shown to improve killing of *Staphylococcus aureus* by phagocytic cells [13]. However, all *in vitro* experiments mentioned above have been performed using statin concentrations between 0.1–50 µM, which greatly exceeds the concentrations present in human blood during statin treatment (1–15 nmol/L) [14].

The aim of the present study was to investigate possible direct antibacterial effect(s) of statins *in vitro* and *in vivo*. Since both epidemiological end experimental data have shown positive effects of statins in respiratory tract infections we here focus on the major respiratory pathogen *Streptococcus pneumoniae* (the pneumococcus).

## Methods

### Chemicals

Simvastatin (lactone and hydroxy acid forms) were purchased from Toronto Research Chemicals. Fluvastatin was obtained from Tocris Bioscience. Pravastatin, Dimethyl sulphoxide (DMSO), Methanol and Mevalonic acid were obtained from Sigma Aldrich.

### Bacterial strains and growth conditions

The serotype 4 *Streptococcus pneumoniae* strain T4 (TIGR4; ATCC BAA-334) [15] and the non-encapsulated laboratory strain R6 were used. Pneumococci were grown overnight on blood agar plates at 37°C and colonies were inoculated into C+Y medium. *Haemophilus influenzae* and *Moraxella catarrhalis* were clinical isolates whose identity were confirmed by 16S RNA sequencing by Clinical Microbiology, Karolinska University Hospital, Solna [16]. *H. influenzae* and *M. catarrhalis* were grown overnight on hematin agar containing X+V-factor and colonies were inoculated in brain heart infusion (BHI-medium) +2% Fildes supplement (Oxoid). Bacteria were grown in 37°C to mid logarithmic phase and subsequently used for antibacterial assays and BioScreen-system experiments.

### Bacterial killing assay

Simvastatin was dissolved in 100% DMSO or 100% methanol. Dilution series (1∶2) in 8 steps were prepared in 100% DMSO with stock solutions (x 40) having concentrations from 10 mM (final concentration in each tube  = 250 µg/ml) to 78 µmol/L (final concentration  = 1.95 µg/ml). Five µL of the simvastatin/DMSO-solution was added to the experimental vials resulting in a final DMSO-concentration of 2.5% in each vial. To this 50 µL bacterial suspension and 145 µL medium was added to obtain a final volume of 200 µL. The tubes were gently shaken and incubated for various times (30 minutes–4 hours) in 37°C. The final bacterial inoculum used for each experiment was 1–5×10^6^ CFU/mL. After the indicated times, 10–100 µL from each tube was diluted in PBS and plated on agar plates. After an over night incubation, numbers of surviving colony forming units were counted.

### BioScreen Experiments

The instrument was purchased from Oy Growth Curves AB, Finland and special honey well plates were used for these experiments (www.bioscreen.fi). In brief, a similar procedure as described above was used for statin and bacterial dilutions and preparations. However, the main difference was that for the antibacterial assays, bacteria were grown to OD600 0.2, whereas the BioScreen experiments were started with bacteria diluted from midlogarithmic phase down to OD600 0.05. This was important in order to synchronize the cultures and to obtain comparable curves between different experiments and between wells. The readout was performed using the software provided by the manufacturer. Data were further processed in Microsoft Excel and Graph Pad Prism.

### *In vivo* experiments on healthy volunteers

The study was approved by the Regional Ethics Committee at Karolinska Institutet, Stockholm (Dnr 2010/ 834-31/3) and written informed consent was obtained from all participants prior to the study. Five healthy volunteers were recruited and given single doses of Simvastatin, 80 mg (n = 3), Fluvastatin, 40 mg (n = 1) and Penicillin (Kåvepenin), 1 g (n = 1). Serum and heparin blood was taken immediately before the tablets were taken and 2 hours after the dose (time of C_max_ for simvastatin) [17]. For Penicillin, the second blood sample was taken 30 minutes after the dose, since this is when C_max_ occurs [18]. Five millilitres of the whole blood were transferred to blood culture flasks and 2×10^6^ CFU of pneumococci strain T4 was added. The flasks were mixed gently and then applied to the BactAlert-system. The read-out in this system is based on a chemical detection system, which set off an alarm when bacterial growth reaches a pre-set level. Whole blood was also used for antibacterial assays where 800 µl of whole blood was mixed with 200 µL of bacterial suspension (6×10^6^ CFU). The tubes were gently mixed during incubation in 37°C and aliquots of 100 µl were drawn after 1, 2, 3 and 4 hours. The aliquots were plated, incubated and counted as described above.

### Measurements of plasma concentrations of fluvastatin and simvastatin

Concentrations of simvastatin (SIM), simvastatin-acid (SIM-OH) and fluvastatin in serum from the healthy volunteers in the in vivo study was measured by a standard liquid chromatography tandem mass spectrometry (LC-MS/MS) method developed for SIM, SIM-OH and fluvastatin as well as for atorvastatin, atorvastatin lactone and rosuvastatin. Sample preparation was based on pH-controlled solid phase extraction followed by evaporation under nitrogen and subsequent reconstitution. Subsequent analysis was performed on a RP-column with a triple quadrupole mass spectrometer as detector. Quantification was calculated on analyte/internal standard peak area ratios with internal standards simvastatin-d6, simvastatin-acid-d6 and atorvastatin-d5 for SIM, SIM-OH and fluvastatin, respectively. Quantitation range for all compounds was 0,05–125 ng/mL with limit of detection at 0,02 ng/mL. This is a recently established method for clinical use in the Clinical Pharmacology Laboratory at Karolinska University Hospital, Stockholm, Sweden. The method is to be described in details elsewhere (Skilving I, et al, manuscript in preparation).

### Statistical analyses

Data was analysed in GraphPad software, version 5.03 for Windows. The non-parametric Mann Whitney U-test or Wilcoxon signed rank test were used as indicated in figure legends. A significance level of p<0.05 was considered as significant.

## Results

### Simvastatin has bactericidal properties against *S. pneumoniae*

**Figure 1:**
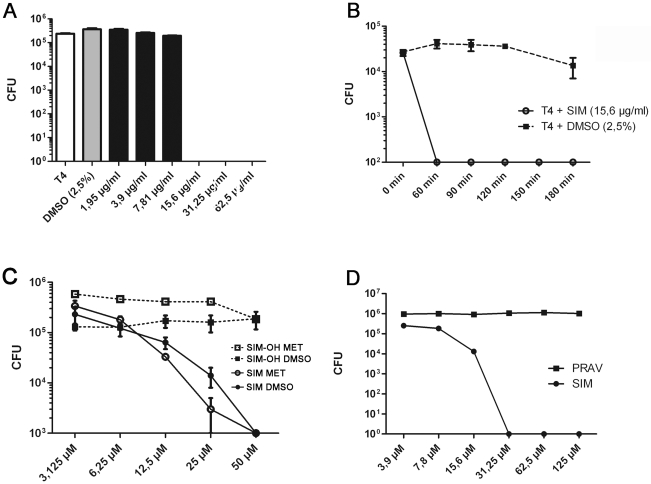
**A**. Simvastatin kills *S. pneumoniae* strain T4 in a concentration dependent manner. Approximately 1×10^5^ CFU of streptococci was incubated for 3–4 hours with various concentrations of simvastatin dissolved in DMSO diluted to a final concentration of 2.5%. The MIC-value was determined to 15.6 µg/ml (36 µmol/L). DMSO (2.5%) alone did not exert any bactericidal effects. The concentrations are calculated and presented in micrograms/ml. Experiments were performed 5 times in triplicates with identical MIC-values. A representative experiment is shown +/- SEM. **B**. Rapid killing of *S. pneumoniae* by simvastatin after 60 minutes. The CFU value for T4 + SIM after 60 minutes is significantly lower compared to baseline (p = 0.03). Experiments were performed 3 times in triplicates with identical kinetics. A representative experiment is shown +/- SEM. **C**. Effect of lactone (SIM) and hydroxy acid (SIM-OH) forms of simvastatin against *S. pneumoniae* T4. Methanol (MET, 2.5%) and DMSO (2.5%) were used solvents for the chemicals, which were investigated at equimolar concentrations for bacterial killing. The CFU value for SIM MET and SIM DMSO at 50 µM is significantly lower than baseline (p = 0.02) and than the CFU values for SIM-OH MET and SIM-OH DMSO at 50 µM (p = 0.03). Experiments were performed 3 times in triplicates with similar results. A representative experiment is shown +/- SEM. **D**. Effects of the hydrophilic pravastatin against *S. pneumoniae*. Pravastatin was compared with simvastatin at equimolar concentrations. The concentrations are calculated and presented in micromolar. The CFU value for SIM at 31.25 µM was significantly lower than baseline (p = 0.02). Experiments were performed 3 times in triplicates with similar results. A representative experiment is shown +/- SEM. Panels A, C and D: Bacteria (1–2×10^5^ CFU) and statins were co-incubated for 3–4 hours, the mixtures were serially diluted and plated. Surviving colonies (CFU) were counted after over night incubation. Mann Whitney U-test was used for statistical analyses.

The antibacterial activity of Simvastatin was investigated using the encapsulated pneumococcal strain TIGR4. 100% killing of viable bacteria was obtained with simvastatin at the concentration 15.6 µg/mL (36 µmol/L) (Fig 1A). The killing of bacteria occurred rapidly and a 4-log reduction occurred in 60 minutes (Fig 1B). Simvastatin is a hydrophobic statin and was dissolved in 2.5% DMSO according to the recommendation of the manufacturer. Since DMSO may have antibacterial activities *per se*, a DMSO-control (2.5%) was included in these experiments. No effect on bacterial killing by DMSO alone was observed during the 180 minutes of incubation during the killing experiments (Fig 1A). To rule out that an intrinsic or synergistic role of DMSO could contribute to our results, simvastatin was dissolved in an alternative solvent (methanol), which produced the same results as the DMSO-dissolved simvastatin (Fig 1C). For these experiments we used the simvastatin-lactone, which is an inactive precursor molecule. To study the potential physiological role of simvastatin as an antibacterial agent, we also obtained the active metabolite simvastatin hydroxy acid (SIM-OH) and repeated the experiments. Interestingly, this metabolite was inactive against pneumococci at equimolar concentrations as the simvastatin lactone (Fig 1C). Two other common statins were also investigated for killing of pneumococci. The hydrophilic pravastatin was dissolved both in water and in DMSO but failed to exhibit any bactericidal activity at concentrations up to 125 µmol/L (Fig 1D). Fluvastatin was also tested and did not exhibit any significant effects at concentrations up to 300 µmol/L (data not shown).

### Simvastatin kills pneumococci independently of HMG-CoA Reductase

**Figure 2:**
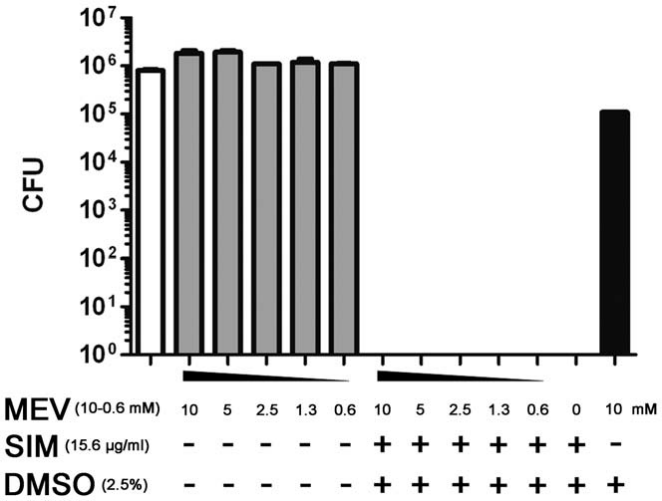
Effects of mevalonate (MEV) on bacterial killing by simvastatin. Different concentrations of mevalonate (0.6 mM–10 mM) were incubated with *S. pneumoniae* alone or together with simvastatin (15.6 µg/ml or 36 µmol/L) or with DMSO (2.5%). Mevalonate could not rescue the simvastatin mediated killing of pneumococci up to concentrations of 10 mM. The empty column designates the positive control (bacteria +buffer), gray columns: experiments with mevalonate (different concentrations) and the black column stands for 10 mM mevalonate +2.5% DMSO. The experiment was performed 3 times in triplicate with similar results, a representative experiment is shown +/- SEM.

Pneumococci express the target enzyme for statins (HMG-CoA reductase) and a deletion of the gene encoding this enzyme has been shown to inhibit bacterial growth [19], [20]. Thus, we used mevalonic acid to rescue blockage of this rate limiting step in cholesterol synthesis. Notably, the presence of 10 mM of mevalonic acid did not rescue simvastatin-mediated killing of pneumococci, which suggest that the killing mechanism does not involve inhibition of bacterial HMG-CoA reductase (Fig 2).

### Simvastatin also kills *M. catarrhalis* but is inactive against *H. influenzae*

**Figure 3:**
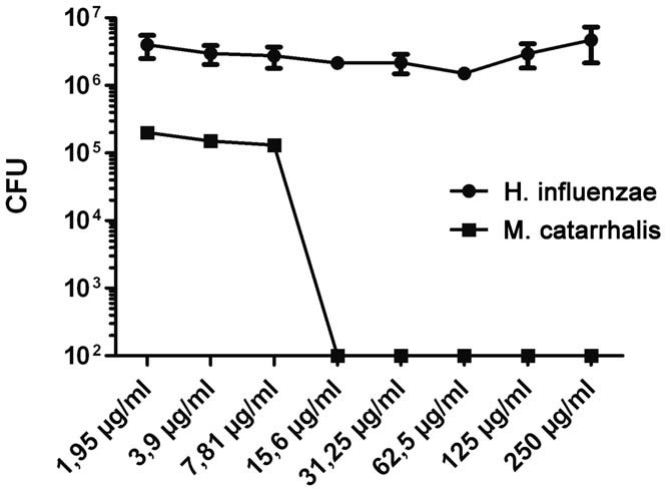
Effects of simvastatin against *H. influenzae* and *M. catarrhalis*. Bacteria (1–2×10^5^ CFU for *M. catarrhalis* and 5×10^6^ CFU for *H. influenzae*) and different concentrations of statins were co-incubated for 3–4 hours, the mixtures were serially diluted and plated. Surviving colonies (CFU) were counted after over night incubation. The CFU counts for *M catarrhalis* at 15.6 µg/ml of simvastatin was significantly lower than baseline (p = 0.04). The experiment was performed 3 times in triplicate with similar results. A representative experiment is shown +/- SEM. Mann Whitney U-test was used for statistical analyses.

The effect of simvastatin was further investigated against two other bacteria responsible for respiratory tract infections, *M. catarrhalis* and *H. influenzae*. A similar effect could be observed for simvastatin against *M catarrhalis* with a MIC-value of 15,6 µg/ml. In contrast, the growth of *H. influenzae* was not affected by simvastatin at concentrations up to 250 µg/ml (600 µmol/L), suggesting a certain specificity with regards to simvastatin-mediated bacterial killing (Fig 3).

### The role of the pneumococcal capsule in statin-mediated killing of pneumococci

**Figure 4:**
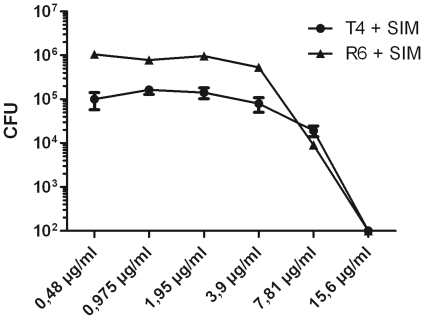
Effect of capsule on simvastatin mediated killing of *S. pneumoniae*. Strains T4 (encapsulated) and R6 (non-encapsulated) were compared for sensitivity of simvastatin mediated killing. Bacteria (1–2×10^5^ CFU) and simvastatin were co-incubated for 3–4 hours, the mixtures were serially diluted and plated. Surviving colonies (CFU) were counted after over night incubation and an identical MIC value (15.6 µg/ml) was obtained for the two strains. The experiment was performed 3 times in triplicate with similar results, a representative experiment is shown +/- SEM.

Since pneumococci constitute a primary pathogen of significant clinical importance and since they were shown to be sensitive to simvastatin, we used this bacterial pathogen for the subsequent experiments. To further investigate the mechanism of killing, the non-encapsulated pneumococcal strain R6 was used. A similar pattern of killing was observed for strains R6 and T4, suggesting that the capsule is not a major determinant for bactericidal effects of simvastatin (Fig 4).

### Growth curves and induction of autolysis

**Figure 5:**
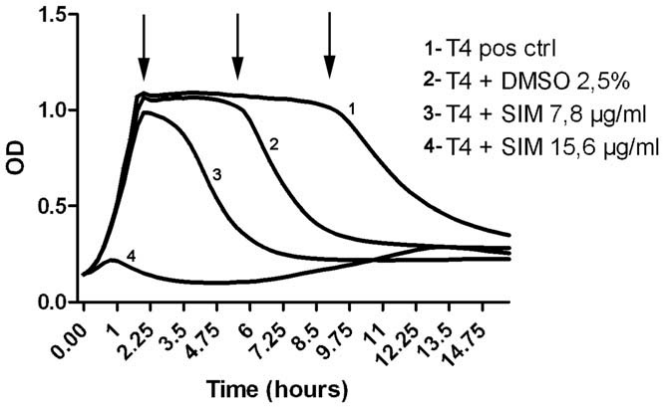
Effects of DMSO and simvastatin on pneumococcal growth. Bacteria were grown to midlogarithmic phase and diluted to OD 0.05. The suspension was co-incubated with simvastatin and DMSO in a BioScreen instrument. Curve 1: positive control (*S. pneumoniae* strain T4). Curve 2: T4 + DMSO (2.5%). Curve 3: T4 + simvastatin at a low concentration below the MIC-value. Curve 4: T4 + simvastatin at MIC-concentration (15.6 µg/ml or 36 µmol/L). Arrows indicate when autolysis was initiated. The experiment was performed 5 times in duplicates with similar results, a representative experiment is shown.

To study the effects of simvastatin on pneumococci in more detail, bacterial growth curves were generated using a BioScreen-system. Bacteria were exposed to simvastatin of different concentrations for 16 hours. A potent growth-inhibiting effect of simvastatin at a concentration of 15,6 µg/ml was observed (Fig 5, curve 4). Unexpectedly, we detected an effect on autolysis by 2.5% DMSO after 5 hours, compared to the natural autolysis in the untreated TIGR4 control that occurred after 9 hours (Fig 5, curves 1 and 2). An autolysis-inducing effect was also observed for simvastatin at the non-bactericidal concentration of 7.8 µg/ml (Fig 5, curve 3). Importantly, the growth curve for simvastatin was different than the curve for DMSO alone, indicating that simvastatin had a specific effect on pneumococci with regards to autolysis (Fig 5, curves 2 and 3).

### Simvastatin and penicillin has an additive effect on autolysis

**Figure 6:**
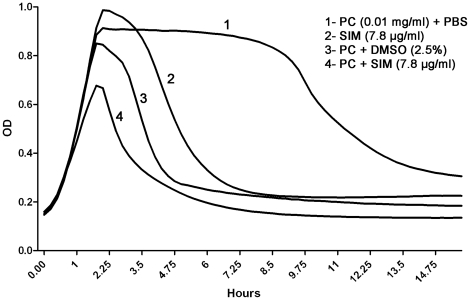
Effects of simvastatin and penicillin (PC-G) on pneumococcal growth. Bacteria was grown to midlogarithmic phase and diluted to OD 0.05. The suspension was co-incubated with simvastatin, DMSO (2.5%) and penicillin in a BioScreen instrument. Curve 1: positive control (*S. pneumoniae* strain T4) + PC-G (0.01 mg/ml). Curve 2: T4 + simvastatin (7.8 µg/ml) in DMSO (2.5%). Curve 3: T4 + PC-G (0.01 mg/ml) + DMSO (2.5%). Curve 4: T4 + PC-G (0.01 µg/ml) and simvastatin (7.8 µg/ml) in DMSO (2.5%). The experiment was performed 3 times in duplicates with similar results, a representative experiment is shown.

The potential synergistic effect between penicillin and simvastatin was investigated using the BioScreen-system. A sub-MIC concentration of penicillin-G was used (0.01 µg/ml), which alone did not affect bacterial growth or induction of autolysis (Fig 6, curve 1). Simvastatin at a non-lethal concentration (7.8 µg/ml) induced autolysis, similarly to previous experiments (fig 5, curve 2). Interestingly, the combination of simvastatin and PC-G was significantly more efficient at inducing autolysis than any of the compounds alone (Fig 6, curve 4). However, PC-G also exerted additive effects together with DMSO on autolysis, although this effect was less pronounced than PC-G used together with simvastatin (Fig 6, curve 3).

### Simvastatin or Fluvastatin do not affect pneumococcal growth in whole blood *ex vivo*

**Figure 7:**
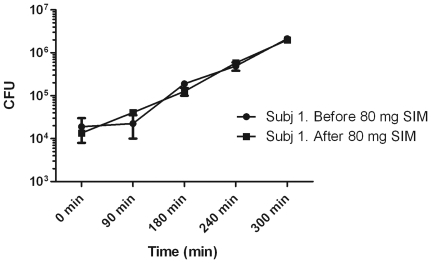
Effects of a single dose simvastatin per os on pneumococcal viability in whole blood. 80 mg simvastatin was given to a healthy volunteer. Whole blood was taken immediately before and 2 hours after intake of the tablet. Bacteria (1×10^4^ CFU) were mixed with the blood and incubated at 37°C. Aliquots were collected at various times and plated. After an over night incubation surviving colonies were counted. There was no statistical significance in CFU counts in blood drawn before or after intake of simvastatin at any timepoint (Wilcoxon signed rank test was used for statistical calculation). The experiment was performed in 3 subjects with similar results. The data from one subject is shown +/- SEM.

The *in vitro* experiments presented above suggested that simvastatin could kill pneumococci at a concentration in the µmol/L-range (MIC = 36 µmol/L). To study a potential *in vivo* role of statins as antibacterial agents we recruited 5 healthy volunteers and gave them maximum dose of Simvastatin (80 mg) or Fluvastatin (40 mg) as single doses. Whole blood was sampled 2 hours later when the serum concentrations were predicted to be on a maximal level (Cmax) and inoculated into blood culture flasks together with pneumococci. Plasma concentrations of fluvastatin was 110.8 nM and for simvastatin 8 nM (acid form, average of 3 individuals) and 19.4 nM (lactone form, average of 3 individuals), respectively (table 1). Whole blood bacterial killing assays were also performed. As a control one study subject ingested 1 gram of Penicillin-V and whole blood was sampled after 30 minutes (Cmax). Intake of 80 mg simvastatin did not affect bacterial growth in whole blood during the first 300 minutes of growth (Fig 7). The blood culture flasks were put into a BactAlert-detection system and bacterial growth was detected after approximately 374–389 minutes for all samples, except in blood derived from study subject 1 after intake of 1 gram PC-V. This blood culture flask had no bacterial growth and was taken out of the system after 5 days (7000 minutes) according to the standard procedure used for clinical work (Table 1).

**Table 1:** Pneumococcal viability in whole blood as measured in a BactAlert system.

Subject no	1	2	3	4	5
Drug	PCV	FLU	SIM	SIM	SIM
**Conc in plasma (Cmax, 2h)**		**110.8** nmol/L	**6.6** nmol/L (SIM-OH) **15.9** nmol/L (SIM)	**13.4** nmol/L (SIM-OH) **32.4** nmol/L (SIM)	**4.1** nmol/L (SIM-OH)**9.9** nmol/L (SIM)
**Time to detection (before)**	374 min	374 min	389 min	374 min	389 min
**Time to detection (after)**	>7000 min	374 min	389 min	389 min	389 min

5 healthy individuals were given either 1 gram penicillin-V (PCV), Fluvastatin (Lescol 40 mg) (FLU) or Simvastatin (80 mg) (SIM) a single doses. Blood was taken immediately before and 30 min (PC-V) and 2 hours (statins) after the intake of the tablets. Concentrations of simvastatin and fluvastatin in serum were measured by LC-MS/MS. The blood was transferred to blood culture flasks, to which pneumococci (6×10^6^ CFU) were added. The flasks were gently mixed and thereafter applied to the BactAlert system. The times indicate “time to detection of bacterial growth” in the system. The flasks are routinely incubated for 5 days (7000 minutes) when they are taken out and discarded.

## Discussion

Epidemiological data suggest that statins may have beneficial effects on mortality during pneumonia [6]. These positive effects have been ascribed a potential anti-inflammatory response mediated by statins. In addition, statins have been demonstrated to exert antibacterial activity *per se* [10], [11]. In this study we investigated whether statins can have antibacterial activity against different respiratory pathogens, including *S. pneumoniae, H. influenzae* and *M. catarrhalis*. Using standard antibacterial assays in liquid broth, we demonstrate MIC-values for simvastatin against *S. pneumoniae* and *M. catarrhalis* of 15 µg/mL (36 mmol/L). Since pneumococci express the target enzyme for statins, HMG-CoA reductase, we investigated whether this enzyme was involved in the observed effect. Notably, mevalonic acid could not rescue the statin mediated killing of pneumococci, underscoring that inhibition of HMG-CoA reductase is not involved in the killing effects. In fact, the true substrate for HMG-CoA reductase, the hydroxy acid form of simvastatin, did not exert any activity, which further emphasizes a non-HMG-CoA reductase dependent effect of simvastatin against pneumococci. We also investigated the hydrophilic compounds fluvastatin and pravastatin, which did not affect bacterial growth up to 300 µmol/L. Thus, a likely mechanism is that the hydrophobic character of simvastatin perturbs the bacterial membrane in a “soap-like” manner, with the final result of bacterial death. Interestingly, *H. influenzae* was not affected by simvastatin, suggesting specificity with regards to the underlying mechanism of statin mediated killing of bacteria. The reason for this statin-resistance of *H. influenzae* remains to be elucidated.

We also performed detailed experiments on statin mediated effects on pneumococci in a BioScreen system, enabling the study of pneumococcal growth curves for up to 16 hours. Membrane perturbation in pneumococci by substances such as penicillin [21], leads to the release of the autolytic enzyme LytA and subsequent degradation of cell wall peptidoglycan (PGN) and autolysis. Interestingly, we noted that simvastatin accelerated the induction of autolysis in pneumococci by 5 hours at sub-MIC doses (Fig 5). We propose that the hydrophobic nature of simvastatin was responsible for the release of LytA and PGN-degradation. Despite the potent effects on autolysis by simvastatin at sub-MIC concentrations, these effects were not translated into reduced bacterial growth. However, since simvastatin is a hydrophobic compound it was dissolved in DMSO, an organic solvent that is widely used in biological experiments and has been described as more or less “inert” to bacterial and human cells. Notably, in 1967 Pottz et al. investigated the effects of DMSO on pneumococcal viability and found that 4% DMSO significantly inhibited bacterial growth and that no growth occurred at 5% DMSO [22]. Here we used DMSO at 2.5% and did not observe any significant effects of DMSO on bacterial viability (Fig 1A and B). Importantly, even though DMSO did not affect bacterial growth it did affect membrane integrity since it had a major impact on autolysis (Fig 5, curve 2). To rule out that all our observed effects could be attributed to DMSO in the system, we also dissolved simvastatin in methanol, and the simvastatin-mediated effect on pneumococcal autolysis could be reproduced, suggesting a true intrinsic effect of simvastatin (data not shown). Nevertheless, our data suggest that DMSO should be used with caution in experiments involving pneumococci, since there are clear effects on autolysis by this compound.

To study a potential synergistic effect between simvastatin and antibiotics, we investigated the effect of simvastatin together with a beta lactam antibiotic (penicillin-G) in the BioScreen assay. Indeed, we could observe a synergistic effect between PC-G and simvastatin on autolysis. This synergy occurred at concentrations below the MIC levels for the respective drug (7.8 µg/mL or 18 µmol/L).

The mean concentration of statins in human serum is only 1–15 nmol/L [14]. In addition, the protein binding of statins in human blood is high, 95–99%, and it is only the free fraction (0.01–0.5 nmol/L) that is pharmacologically active [14]. Thus, the MIC-value for simvastatin (15 µg/L or 36 µmol/L) and the concentration giving synergistic effect on autolysis with PCG (7.8 µg/L or 18 µmol/L) is approximately 1000-fold higher than what can be achieved in humans, which strongly argue against any relevant antibacterial effect of statins *in vivo* with or without antibiotics.

However, it could be speculated that statins affect the killing capacity of immune cells in the blood, as proposed by Chow et al [13]. Thus, we conducted a small pilot-study where healthy volunteers were given a single dose of simvastatin or fluvastatin and blood were collected before and after statin intake. The positive control was a healthy volunteer taking penicillin. We compared the growth of bacteria in blood before and after statin intake. By this approach we studied statins at physiological concentrations *in vivo* without the solvents (DMSO or methanol) that are present in the *in vitro* experiments. Importantly, bacterial growth could be detected in all blood flasks at approximately the same time (374 minutes), whereas the blood flasks from the single individual taking penicillin did not yield any growth even after 5 days, indicating rapid bacterial killing. Hence, a single dose of statin does not result in sufficient concentrations for pneumococcal killing in whole blood. Moreover, our data suggest that a single dose of statins does not improve monocyte- or neutrophil-mediated killing of pneumococci in whole blood.

The main limitation of our study is that we only studied a single dose of statins and we cannot rule out any long term antibacterial effect of statins in patients taking this drug. Nevertheless, since many mechanistic studies have been performed using non-physiological concentrations of statins [14] and that the epidemiological evidence may be flawed by publication bias [6], randomized controlled trials of statin treatment during infections are highly warranted.
